# Neonatal Orally Administered Zingerone Attenuates Alcohol-Induced Fatty Liver Disease in Experimental Rat Models

**DOI:** 10.3390/metabo13020167

**Published:** 2023-01-23

**Authors:** Bernice Asiedu, Busisani Wiseman Lembede, Monica Gomes, Abe Kasonga, Pilani Nkomozepi, Trevor Tapiwa Nyakudya, Eliton Chivandi

**Affiliations:** 1 School of Physiology, Faculty of Health Sciences, University of the Witwaterstrand, 7 York Street, Parktown, Johannesburg 2193, South Africa; 2 Department of Physiology, School of Medicine, Faculty of Health Sciences, University of Pretoria, Private Bag X323, Gezina, Pretoria 0031, South Africa; 3 Department of Human Anatomy and Physiology, Faculty of Health Sciences, University of Johannesburg, Corner Beit and Siemert Street, Doornfontein, Johannesburg 2094, South Africa

**Keywords:** alcohol-induced fatty liver disease, zingerone, peroxisome proliferator activator receptor-alpha (*PPAR-α*), sterol regulatory element binding protein 1c (*SREBP1c*), macrosteatosis

## Abstract

Alcohol intake at different developmental stages can lead to the development of alcohol-induced fatty liver disease (AFLD). Zingerone (ZO) possess hepato-protective properties; thus, when administered neonatally, it could render protection against AFLD. This study aimed to evaluate the potential long-term protective effect of ZO against the development of AFLD. One hundred and twenty-three 10-day-old Sprague–Dawley rat pups (60 males; 63 females) were randomly assigned to four groups and orally administered the following treatment regimens daily during the pre-weaning period from postnatal day (PND) 12–21: group 1—nutritive milk (NM), group 2—NM +1 g/kg ethanol (Eth), group 3—NM + 40 mg/kg ZO, group 4—NM + Eth +ZO. From PND 46–100, each group from the neonatal stage was divided into two; subgroup I had tap water and subgroup II had ethanol solution as drinking fluid, respectively, for eight weeks. Mean daily ethanol intake, which ranged from 10 to 14.5 g/kg body mass/day, resulted in significant CYP2E1 elevation (*p* < 0.05). Both late single hit and double hit with alcohol increased liver fat content, caused hepatic macrosteatosis, dysregulated mRNA expression of *SREBP1c* and *PPAR-α* in male and female rats (*p* < 0.05). However, neonatal orally administered ZO protected against liver lipid accretion and *SREBP1c* upregulation in male rats only and attenuated the alcohol-induced hepatic *PPAR-α* downregulation and macrosteatosis in both sexes. This data suggests that neonatal orally administered zingerone can be a potential prophylactic agent against the development of AFLD.

## 1. Introduction

Alcohol liver disease (ALD) develops from prolonged excessive alcohol consumption. Studies show that genetic predisposition contributes substantially to alcohol use disorder [1,2]. Others show that exposure to alcohol in utero trigger epigenetic modifications that also may play a role in mediating alcohol use disorders [3,4]. Numerous studies indicate that the intrauterine environment can set either a healthy or diseased trajectory for offspring in the future; a phenomenon described as the developmental origin of health and disease [5,6]. We hypothesized that ALD may develop based on this concept [7]. Human and preclinical studies indicate that prenatal alcohol exposure (PAE) predisposes offspring to increased alcohol consumption in adolescence and adulthood [8,9]. Consumption of alcohol in adolescence has been shown to cause use disorders and abuse, which may result in the development of ALD [10,11]. Hence, early-life exposure to alcohol and/or alcohol consumption in adolescence may be a significant risk factor for ALD. Therefore, the combination of early-life alcohol exposure and alcohol consumption in adolescence constitute a double-hit effect for the development of ALD. Although the suckling growth phase is equally susceptible to neuroplasticity as the prenatal phase [12], its impact on alcohol consumption in adolescence and the subsequent development of ALD have not been thoroughly explored.

Chronic liver disease affects about 10% of the world’s human population and, its mortal end-stage generally follows cirrhosis and liver cancer [13]. Varied factors characterize liver disease as the fourth to the fifth cause of death worldwide [13]. Non-alcoholic fatty liver disease ranks first contributing up to 40% of liver diseases and hepatitis B and C viruses and alcohol overconsumption contribute 30%, 15% and 11%, respectively [13]. Alcohol abuse ranks third to smoking and hypertension as a cause of preventable death [14]. Alcohol-induced liver disease accounts for 20% to 50% of the prevalence of liver cirrhosis [15]. Alcohol consumption results in liver damage on a spectrum, from simple steatosis to hepatitis with fibrosis [16,17]. The development of a fatty liver is the earliest response to heavy (>40 g ethanol/day) and/or chronic consumption of alcohol and this occurs in about 90% of heavy drinkers [18]. However, only 8–20% of those that develop fatty liver progress to severe forms of liver disease [18].

Steatosis, which is categorized as either macro- and or microsteatosis, occurs when more than 5% fat constitutes liver tissue resulting in hepatocytes becoming distended with lipids [19]. Hepatic macrosteatosis is typified by the presence of a large fat droplet that pushes the nucleus to the periphery of the hepatocyte [19,20], while with microsteatosis, many small, less than 1 μm in diameter, cytoplasmic lipid droplets give the hepatocyte a foamy-like appearance but with the nucleus remaining in the middle of the cell [19,20]. There are two variants of macrosteatosis: large and small droplet macrosteatosis [21]. In the former, a large unilocular lipid droplet fills up the hepatocyte and pushes the nucleus to the periphery and in the latter are multilocular lipid droplets that occupy less than half of the cytoplasm with the nucleus remaining intact [21]. In the progression of steatosis, small lipid droplets coalesce into large fat droplets [22]. Studies show that macrosteatosis is common [23], but microsteatosis is rare in AFLD [22]. Macrosteatosis has a good prognosis with rare progression to fibrosis [24].

Although the pathogenesis of ALD is not entirely understood, it is known to stem from the toxic effects of ethanol and its metabolite acetaldehyde (AA), which mediate increased intestinal permeability, changes to the gut microbiome and an inflammatory response to cellular injury [25]. In vivo, alcohol dehydrogenase (ADH) metabolises ethanol to AA. The metabolism of ethanol involves the upregulation of sterol regulatory element-binding transcription factor 1 c (*SREBP-1c*), which upon interacting with lipid droplets membrane proteins, promotes the formation of lipid droplets in hepatocytes resulting in the development of steatosis [26]. An increase in the reduced nicotinamide adenine dinucleotide (NADH) to oxidised nicotinamide adenine dinucleotide (NAD^+^) ratio inhibits β-oxidation and activates triglyceride synthesis [25], which increases the quantity of fat in hepatocytes. Additionally, the inhibition of the transactivation activity and DNA-binding of peroxisome activator receptor α (*PPAR-α*) by AA results in inhibiting β-oxidation [26]. 

The metabolism of low to moderate amounts of consumed alcohol induces the ADH system, but when excess alcohol is consumed, its metabolism induces the cytochrome P450 E21 (CYP2E1) system [27]. Induction of the CYP2E1 generates excessive amounts of reactive oxygen species that then mediate lipid peroxidation [28]. The by-products of lipid peroxidation, for example, malondialdehyde and 4-hydroxynonenal, activate adaptive immunity [25] and the intrinsic apoptotic pathways [29]. Activation of these pathways result in hepatic neutrophil infiltration and liver inflammation via the myeloid differentiation primary response gene 88 (MyD88) independent signalling pathway [25]. Recruitment of the MyD88 mediates the activation of the pro-inflammatory transcription factor, nuclear factor kappa β (NF-Kβ) and its downstream inflammatory cytokines; tumour necrosis factor-alpha (TNF-α), Interleukin (IL)-6, IL-10 and IL-1β [30,31] that contribute to hepatocellular damage.

Several pharmacological agents have proven effective as potential prophylaxes in managing adverse effects induced by exposure to toxic substances during periods of developmental plasticity [32,33,34]. Zingerone has been reported to possess antisteatotic properties that can protect against the development of fatty liver via the activation of AMP-activated protein kinase (AMPK) [33,35]. Furthermore, oral supplementation of 40 mg/kg body wt of zingerone via intragastric intubation to alcohol-fed Wistar rats protected against hepatoxicity [36]. We therefore determined the prophylactic potential of zingerone to protect against the development of alcohol-induced fatty liver disease using rat models.

## 2. Materials and Methods

### 2.1. Study Setting and Animal Use Ethical Clearance

This study was conducted at the Wits Research Animal Facility (WRAF) of the University of the Witwatersrand, Johannesburg, South Africa. This study complied with accepted laboratory animal use and care stipulated in the South African National standard (SANS: 10386:2008) and the animal protection act 1962, Act No. 71. Ethical clearance for the experiment (ethical clearance certificate number: 2019/10/57B) was granted by the Animal Research Ethics Committee of the University of the Witwatersrand.

### 2.2. Animals and Animal Management

The study used one hundred and twenty-three 10-day-old suckling male and female Sprague–Dawley rat pups (60 males; 63 females) from dams with 8 to 12 rat pups. During the suckling period, from postnatal day (PND) 1 to 21, the rat litter were housed with their respective dams in acrylic cages at the WRAF. At weaning from PND 21 to PND 100, the rat pups were each individually housed in acrylic cages and allowed ad libitum access to feed and tap water. The room temperature was maintained at 24 ± 2 °C. A 12:12 h dark-and-light cycle (with lights on at 07:00 h) was kept throughout the experimental period.

### 2.3. Experimental Design

**Figure 1:**
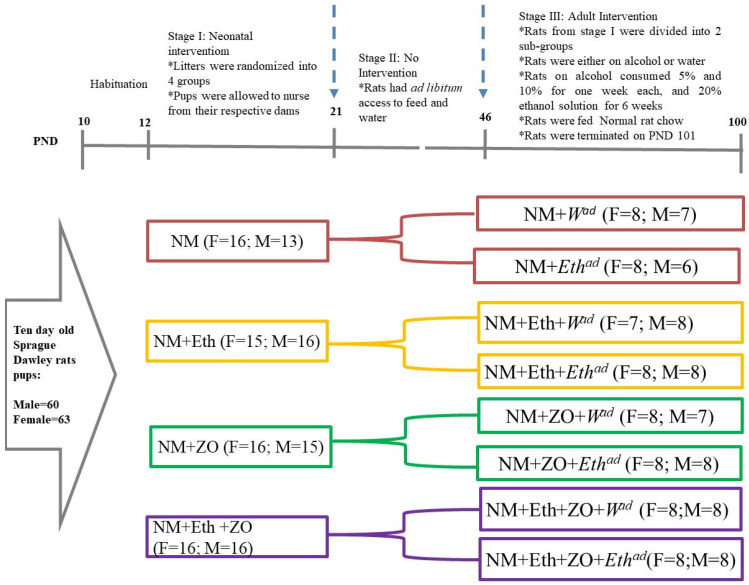
Study experimental design: control (NM + *W^ad^*)—neonatally gavaged with nutritive milk (NM) and drank plain drinking water (*W^ad^*) at stage III; late single hit (NM + *Eth^ad^*)—neonatally gavaged with NM and drank ethanol (*Eth^ad^*) solution at stage III; early single hit (NM + Eth + *W^ad^*)—neonatally gavaged with ethanol in nutritive milk and drank plain drinking water (*W^ad^*) at stage III; double hit (NM + Eth + *Eth^ad^*)—neonatally gavaged with Eth in nutritive milk and drank *Eth^ad^* at stage III; zingerone alone (NM + ZO + *W^ad^*)—neonatally gavaged with ZO in nutritive and drank plain drinking water (*W^ad^*) at stage III; early single hit with zingerone (NM + Eth + ZO + *W^ad^*)—neonatally gavaged with NM, Eth, ZO and drank plain drinking water (*W^ad^*) at stage III; late single hit with zingerone (NM + ZO + *Eth^ad^*)—neonatally gavaged with NM and ZO and drank *Eth^ad^* at stage III; double hit with zingerone (NM + Eth + ZO + *Eth^ad^*)—neonatally gavaged with NM, Eth and ZO and drank *Eth^ad^* at stage III; F—female; M—male; NM—nutritive milk; Eth—ethanol; ZO—zingerone; W—water; *n* = 6–8 per treatment group.

This interventional study comprised three stages (Figure 1): intervention during the neonatal growth phase, a growing out phase with no intervention and intervention during the adult growth phase (Figure 1). At the neonatal stage, 123 10-day-old rat pups (60 males; 63 females) following a 2-day habituation period were then randomly assigned to four groups and administered different treatment regimens as follows: Group I: nutritive milk (NM); Group II: NM + 1 g/kg body mass ethanol (NM + Eth); Group III: NM+ 40 mg/kg body mass of ZO (NM + ZO); Group IV: NM + Eth + ZO. Interventions were administered from PND 12–21. Ethanol and zingerone dosage have previously been used in rat pups [35,37,38]. Nutritive milk was used as the vehicle for administration of the interventions. During the second stage (PND 22–45), the weaned rats were allowed to grow to adulthood without any intervention but had ad libitum access to normal rat chow and plain drinking water.

The intervention during adulthood started from PND 46 and continued to PND 100. During this intervention, rats from each group at the neonatal stage were divided into two subgroups; rats in subgroup I had plain drinking water and their counterparts in subgroup II had ethanol as drinking fluids for eight weeks (Figure 1). The rats were adapted to incremental ethanol solution initially at 5% (*v*/*v*) for one week, then 10% (*v*/*v*) for another week, and 20% (*v*/*v*) ethanol solution for the remaining six weeks per the protocol described by Ojeda et al., 2008 [39]. Rats have a natural aversion to alcohol and prefer ethanol solutions at lower concentrations [40]. Hence, they reduce their intake volumes as the ethanol concentration increases [40]. Thus, incremental ethanol at 5 and 10% was used to prime them to the taste of alcohol and prevent decreased intake at 20% ethanol concentration. The amount of ethanol consumed weekly by each was measured and computed in g/100 g body mass using the “Tables for determining grams values of ethanol solution” previously described [41].

The rats that were orally gavaged with alcohol during the neonatal growth phase and had ethanol solution as drinking fluid in adulthood had a double hit with alcohol, and those that were gavaged with alcohol during the neonatal growth phase only had an early single hit with alcohol. The rats in the subgroups that received alcohol solution as a drinking fluid only during adulthood had a late single hit.

### 2.4. Terminal Procedure

On PND 101, following an overnight fast, the terminal body masses of the rats were measured using an electronic balance (Snowrex, Johannesburg, South Africa). The rats were then euthanised with 200 mg/kg of sodium pentobarbital (Eutha-naze^®^, Bayer, Johannesburg, South Africa) via intraperitoneal injection and cut open by a midline incision on the abdomen and thorax. Blood was drawn via cardiac puncture using an 18 G syringe into heparinised tubes and then centrifuged (Senova NovaFuge centrifuge, Shanghai, China) at 3000× *g* for 15 min. Plasma was then collected and stored at −80 °C for further biochemical assays.

Each rat’s liver was carefully dissected from the abdominal cavity and weighed on an electronic scale (Presica 310 M, Presica Instruments, Dietikon, Switzerland). The liver was then divided into four parts. One part was rinsed in cold saline and stored at −20 °C to determine hepatic thiobarbituric acid (TBARS). A sample from the right lobe of each liver was immersion fixed in 10% phosphate-buffered formalin solution (Merck, Johannesburg, South Africa) for histological analysis. Another part of the liver was stored in sealed ziplock plastic bags at −20 °C for total fat content determination, and the last portion was placed in RNA*later* solution and kept at −80 °C for molecular analysis.

### 2.5. Computation of the Hepatosomatic Index

The hepatosomatic index was computed by dividing the mass of each liver by the respective terminal body mass of each rat and expressed as a percentage (%).

### 2.6. Determination of Hepatic Lipid Peroxidation

#### 2.6.1. Liver Tissue Homogenisation

The liver sample (100 mg) was homogenised in 10 mL of phosphate buffer (0.1 M, pH = 7.4) with an ultra turrax homogeniser (T-25 basic, Janke & Kunkel Ultra Turrax, Germany). The resultant homogenate was centrifuged at 3000× *g* for 15 min at 4 °C. The resultant supernatant was used to determine hepatic lipid peroxidation by measuring the supernatant’s TBARS concentration.

#### 2.6.2. Determination of Peroxidation in the Liver

The liver TBARS concentration was estimated using the method described by Niehaus and Samuelsson (1968) [42]. Briefly, 2 mL of the supernatant from the liver sample homogenate was diluted with distilled water in a 1:1 ratio. 2.0 mL of the working reagent ((thiobarbituric acid (TBA)–trichloroacetic acid (TCA)–hydrochloric acid (HCL)) in a ratio of 1:1:1) was then added to the diluted supernatant. The mixture was boiled in a water bath for 15 min, allowed to cool on ice for 5 min, and then centrifuged (Senova NovaFuge centrifuge, Shanghai, China) at 3000× *g* for 5 min at room temperature. The absorbance of the supernatant obtained was read on a spectrophotometer (Beckman Coulter, CA, USA) at 532 nm.

### 2.7. Determination of Liver Lipid Content

The total liver lipid content was determined using the soxhlet extraction method as described by AOAC (2005; method number 920.39) using petroleum ether as the solvent.

### 2.8. Determination of Liver Histomorphometry

Liver tissue samples preserved in 10% phosphate-buffered formalin were processed with an automated tissue processor (Microm STP 120 Thermo Scientific, Waltham, MA, USA), embedded in paraffin wax and sectioned at 3 µm using a microtome (Leica instruments GmbH, (Pty) Ltd., Wetzlar, Germany) for histological analysis. The sections were stained with haematoxylin and eosin (H&E) using a Gemini AS slide stainer coupled to a Clearvue cover slipper (Fisher Scientific, Waltham, MA, USA). The stained liver sections were viewed under an Olympus BH2-RFCA microscope (Olympus Corporation, Tokyo, Japan) coupled to an Olympus XC 10 HD camera (Olympus Corporation, Tokyo, Japan) for histological assessment and image capture.

### 2.9. Determination of Surrogate Plasma Biomarkers of Liver Function

Plasma activities of aspartate aminotransferase (AST) and alanine aminotransferase (ALT) were determined using an automated clinical chemistry analyser (IDEXX VetTest^®^ Clinical Chemistry Analyser, IDEXX Laboratories Inc., Westbrook, ME, USA) according to the manufacturer’s instructions. The calibrated auto-analyser performed the analysis on pre-loaded disks for AST and ALT using 10 μL of plasma.

### 2.10. Determination of Plasma CYP2E1, TNF-α and IL-6 Concentration 

Rat-specific ELISA kits (Elabscience^®^, Rat CYP2E1, TNF-α, IL-6 ELISA kit, Wuhan, Hubei Province, China) were used to determine the plasma CYP2E1, tumour necrosis factor-alpha (TNF-α) and interleukin-6 (IL-6) concentration following the manufacturers’ instructions. The test employed a sandwich ELISA principle. The optical density of the resulting reactions was measured at 450 nm on a microplate reader (Thermo Fisher Scientific Inc., Waltham, MA, USA), and the sample concentrations were extrapolated from the standard curve.

### 2.11. Determination of Hepatic Gene Expression 

#### 2.11.1. RNA Extraction and cDNA Synthesis

Liver tissue (50 mg) was finely ground with a mortar and pestle, and the RNA was extracted using Aurum^™^ Total RNA Mini Kit (BioRad, Hercules, CA, USA). The RNA quantity was assessed by measuring the absorption at 260 nm and the purity by the 260/280 nm absorbance ratio using the NanoDrop lite spectrophotometer (Thermofisher Scientific, Johannesburg, South Africa). The volume of RNA needed to make a final concentration of 0.5 μg was calculated and synthesised to complementary DNA (cDNA) with LunaScript supermix (Inqaba biotec, Johannesburg, South Africa). Nuclease-free water was added to make a final volume of 20 μL. The preparation was gently mixed and incubated at 25 °C for 2 min, 55 °C for 10 min, and 95 °C for 1 min on the thermal cycler (PxE0.2, Thermo Fisher Scientific, Waltham, MA, USA). The cDNA samples were then stored at −20 °C until further use.

#### 2.11.2. Reverse Transcriptase Polymerase Chain Reaction (RT-PCR) Analysis

Real-time PCR was performed using LunaScript master mix (Inqaba biotec, Johannesburg, South) with the master mix and primers mixed according to the manufacturer’s protocol. The primers used are provided in Supplementary Table S1. Complementary DNA was diluted to 1:20 ratio with nuclease-free water. The prepared mix was added to appropriate wells in 96-well plates (Roche, Johannesburg, South Africa). The cDNA template was added last and the plates were sealed with optical adhesive covers. Quantitative real-time PCR (qRT-PCR) was measured on the LightCycler 96 (Roche diagnostics, Basel, Switzerland) following thermal cycling conditions of the manufacturers’ protocol. Relative gene expression was analysed using the −2^ΔΔCT^ method. Gene expression was normalised to the mRNA expression of *beta-actin*.

#### 2.11.3. Statistical Analysis

GraphPad Prism 8 software (Graph-Pad Software Inc., San Diego, CA, USA) was used to analyse data. Data are expressed as mean ± standard deviation. A one-way ANOVA was used to analyse multiple-group data, followed by mean comparison using the Tukey post hoc test for parametric data. The Kruskal–Wallis test was used to analyse multiple group nonparametric data, followed by mean comparisons by the Dunns post hoc test for nonparametric. Statistical significance was considered when *p* < 0.050. 

## 3. Results

### 3.1. Effect of Neonatal Orally Administered Zingerone on Ethanol Consumption in Adult Rats

**Figure 2:**
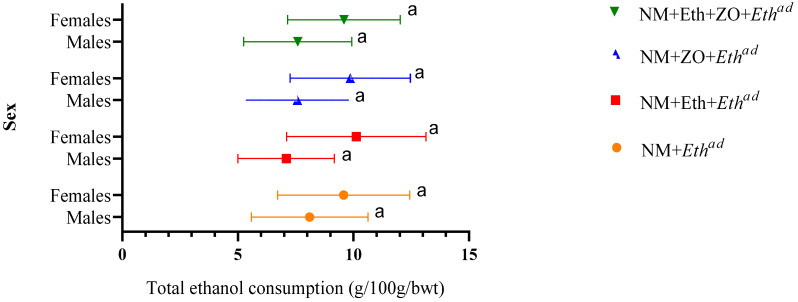
Effect of neonatal orally administered zingerone on ethanol consumption. Data are presented as mean ± sd. ^a,a^ = Line graphs with similar letters do not differ significantly at *p* > 0.05. Control (NM + *W^ad^*)—neonatally gavaged with nutritive milk (NM) and drank plain drinking water (*W^ad^*) at stage III; late single hit (NM + *Eth^ad^*)—neonatally gavaged with NM and drank ethanol (*Eth^ad^*) solution at stage III; early single hit (NM + Eth + *W^ad^*)—neonatally gavaged with ethanol in nutritive milk and drank plain drinking water (*W^ad^*) at stage III; double hit (NM + Eth + *Eth^ad^*)—neonatally gavaged with Eth in nutritive milk and drank *Eth^ad^* at stage III; zingerone alone (NM + ZO + *W^ad^*)—neonatally gavaged with ZO in nutritive and drank plain drinking water (*W^ad^*) at stage III; early single hit with zingerone (NM + Eth + ZO + *W^ad^*)—neonatally gavaged with NM, Eth, ZO and drank plain drinking water (*W^ad^*) at stage III; late single hit with zingerone (NM + ZO + *Eth^ad^*)—neonatally gavaged with NM and ZO and drank *Eth^ad^* at stage III; double hit with zingerone (NM + Eth + ZO + *Eth^ad^*)—neonatally gavaged with NM, Eth and ZO and drank *Eth^ad^* at stage III; NM—nutritive milk; Eth—ethanol; ZO—zingerone; W—water; ^ad^—adult treatment; *n* = 6–8 per treatment group.

Figure 2 shows the weekly mean ethanol intake of the rats. In male and female rats, early and late single hit as well as double hit of alcohol alone or together with neonatal zingerone did not affect ethanol consumption in adulthood (*p* > 0.05). Weekly ethanol intake ranged from 7.02 ± 2.09 g/100 body mass to 10.12 ± 3.01 g/100 g body mass (Supplementary Table S2). Male rats that had a late single hit with alcohol had the highest ethanol intake, while female rats that had a double hit with alcohol had the highest ethanol intake among counterpart male and female rats that consumed ethanol solution in adulthood.

### 3.2. Effect of Neonatal Orally Administered Zingerone on Hepatosomatic Index

Treatment regimens had no effect on the absolute liver masses and hepatosomatic indices of the male rats (*p* > 0.05 vs. control; Table 1). The absolute liver mass and hepatosomatic indices of the female rats in each treatment group did not differ from the control counterparts (*p* > 0.05 vs. control; Table 1). Female rats that had an early single hit with alcohol had a significantly increased absolute liver mass compared to counterparts that had zingerone orally administered during the neonatal growth phase in combination with either a late single hit and or a double hit with alcohol (*p* = 0.023, NM + Eth + *W^ad^* vs. NM + ZO + *Eth^ad^*; *p* = 0.049, NM + Eth + *W^ad^* vs. NM + Eth + ZO + *Eth^ad^*). When adjusted to the body mass, the hepatosomatic index of the female rats was not significantly different (*p* > 0.05) compared to the other treatment groups.

### 3.3. Effect of Neonatal Orally Administered Zingerone on Liver Fat Content

**Figure 3:**
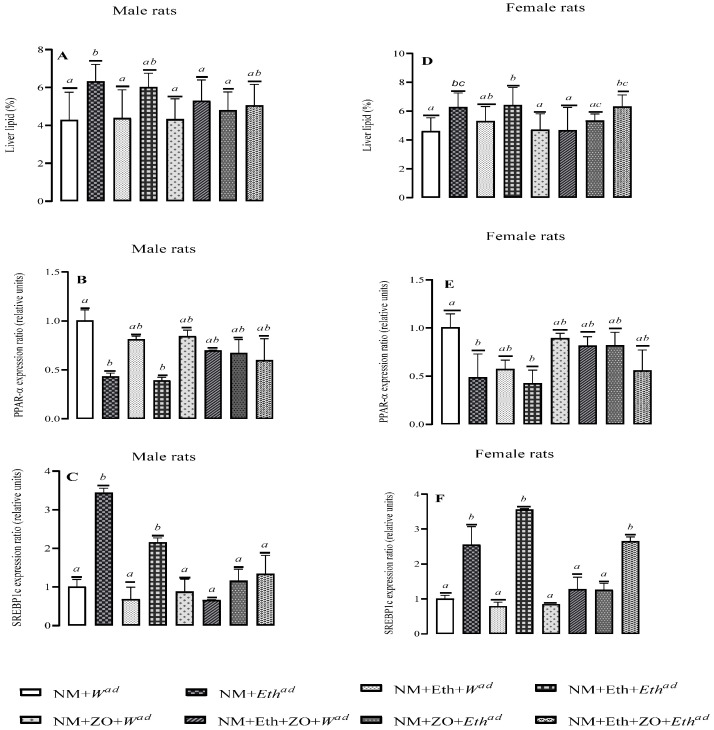
Effect of neonatal orally administered zingerone on liver lipid (**A**,**D**); PPAR-α and (**B**,**E**) SREBP1c (**C**,**F**) gene expression in male and female rats drinking alcohol in adulthood. Data are presented as mean ± sd. ^a,b^ = Bars with different letters differ significantly at *p* < 0.05. Control (NM + *W^ad^*)—neonatally gavaged with nutritive milk (NM) and drank plain drinking water (*W^ad^*) at stage III; late single hit (NM + *Eth^ad^*)—neonatally gavaged with NM and drank ethanol (*Eth^ad^*) solution at stage III; early single hit (NM + Eth + *W^ad^*)—neonatally gavaged with ethanol in nutritive milk and drank plain drinking water (*W^ad^*) at stage III; double hit (NM + Eth + *Eth^ad^*)—neonatally gavaged with Eth in nutritive milk and drank *Eth^ad^* at stage III; zingerone alone (NM + ZO + *W^ad^*)—neonatally gavaged with ZO in nutritive and drank plain drinking water (*W^ad^*) at stage III; early single hit with zingerone (NM + Eth + ZO + *W^ad^*)—neonatally gavaged with NM, Eth, ZO and drank plain drinking water (*W^ad^*) at stage III; late single hit with zingerone (NM + ZO + *Eth^ad^*)—neonatally gavaged with NM and ZO and drank *Eth^ad^* at stage III; double hit with zingerone (NM + Eth + ZO + *Eth^ad^*)—neonatally gavaged with NM, Eth and ZO and drank *Eth^ad^* at stage III; ^ad^—adult treatment; NM—nutritive milk; Eth—ethanol; ZO—zingerone; W—water; n = 6–8 per treatment group for liver lipids. For lipid regulatory genes, *PPAR-α* and *SREBP1c*; *n* = 2–3 rats per treatment group.

Treatment regimens significantly affected the liver fat content of male and female rats (*p* = 0.006, (males): *p* = 0.008, (females) Figure 3). A late single hit with alcohol significantly increased (*p* = 0.039; Figure 3A) liver fat content of male rats compared to control counterparts but a combination of neonatal orally administered zingerone with a late single hit decreased liver fat content compared to male counterparts that only had a late single alcohol hit (*p* = 0.036, NM + ZO + *Eth^ad^* vs. NM + *Eth^ad^*, Figure 3A). An early single and double hit with alcohol alone or together with neonatal administered zingerone had no effect on liver fat content of male rats (*p* > 0.05; Figure 3A).

A late single and double hit with alcohol significantly increased liver fat content of female rats (*p* = 0.045, NM + *Eth^ad^* vs. NM + *W^ad^*; *p* = 0.023, NM + Eth + *Eth^ad^* vs. NM + *W^ad^*, Figure 3D). Neonatal orally administered zingerone in combination with either a single and or double alcohol hit resulted in similar liver fat content with that of control counterparts (*p* = 0.858, NM + ZO + *Eth^ad^* vs. NM + *W^ad^*; *p* = 0.067, NM + Eth + ZO + *Eth^ad^* vs. NM + *W^ad^*; Figure 3D). The liver fat content of female rats that had a combination of neonatal zingerone and double alcohol hit was similar to that of counterparts that had a late single or double alcohol hit (*p* > 0.05, Figure 3D).

### 3.4. Effect of Neonatal Orally Administered Zingerone on Hepatic Histomorphometric Changes

**Figure 4:**
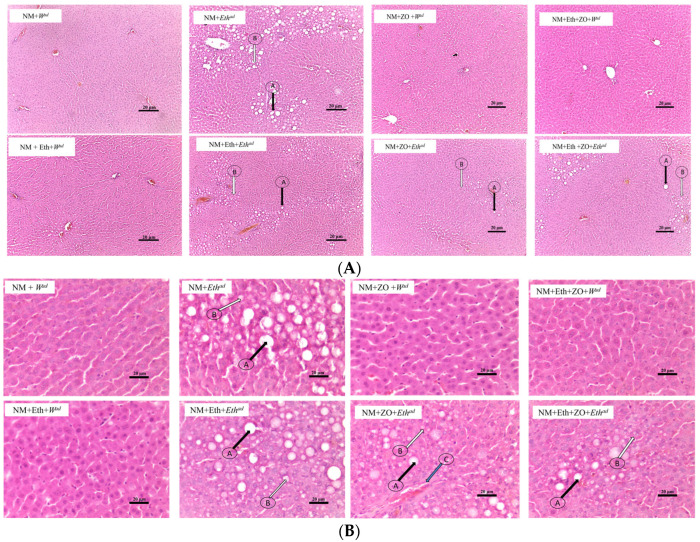
Photomicrographs of male (**A**) liver sections stained with H&E. Magnification X10 arrows Photomicrographs of male (**B**) liver sections stained with H&E. Magnification X40 arrows A = large droplet macrosteatosis; arrows B = small droplet macrosteatosis; arrows C = inflammatory cell infiltrate; arrow D = microsteatosis. NM—nutritive milk; Eth—ethanol; ZO—zingerone; W—water; ^ad^—adult treatment.

**Figure 5:**
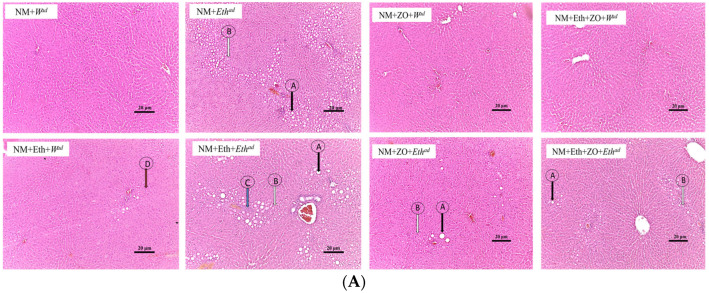

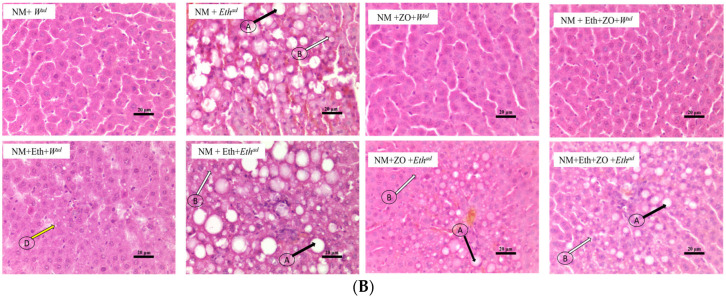
(**A**) Photomicrographs of male female liver sections stained with H&E. Magnification X10 (**B**) Photomicrographs of male female liver sections stained with H&E. Magnification X40 arrows A = large droplet macrosteatosis; arrows B = small droplet macrosteatosis; arrows C = inflammatory cell infiltrate; arrow D = microsteatosis. NM—nutritive milk; Eth—ethanol; ZO—zingerone; W—water; ^ad^—adult treatment.

Figure 4A and Figure 5A show the histomorphometric changes of the liver sections at X10. This is to provide an overview of the treatment effect. Figure 4B and Figure 5B is produced at X40 for differentiation of the steatotic cells. Hepatic lobules of male and female rats that consumed plain tap water (NM + *W^ad^*, NM + Eth + *W^ad^*, NM + ZO + *W^ad^* and NM + Eth + ZO + *Eth^ad^*) contained regularly arranged hepatocytes radiating from the central vein and had no visible hepatic steatosis in the control rats. An early single hit with alcohol (NM + Eth + *W^ad^*) resulted in microsteatosis in female rats (Figure 5B; arrow D), which was not observed in the female rats that had an early single hit alcohol in combination with neonatal zingerone (Figure 5B). The late single hit (NM+ *Eth^ad^*) and double hit (NM + Eth + *Eth^ad^*) with alcohol resulted in small and large droplet steatosis (Figure 4 and Figure 5; arrow A & B) in male and female rats. A combination of neonatal zingerone with the late single hit (NM + ZO + *Eth^ad^*) and double hit (NM + Eth + ZO + *Eth^ad^*) of alcohol resulted in relatively less severe small and large droplet steatosis (Figure 4 and Figure 5).

### 3.5. Effect of Neonatal Orally Administered Zingerone on Lipid Regulatory Genes

Treatment regimens had a significant (*p* < 0.05) effect on the mRNA expression level of *PPAR-α* of both male and female rats. Both late single and double hit with alcohol significantly decreased (*p* = 0.014, NM + *Eth^ad^* vs. NM + *W^ad^*; *p* = 0.0009, NM + Eth + *Eth^ad^* vs. NM + *W^ad^*, Figure 3B) *PPAR-α* expression levels in male rats and they also significantly decreased (*p* = 0.040, NM + *Eth^ad^* vs. NM + *W^ad^*; *p* = 0.019, NM + Eth + *Eth^ad^* vs. NM + *W^ad^* Figure 3B) that of females relative to control. Neonatal orally administered ZO in combination with either a late single or double alcohol hit had no effect on *PPAR-α* expression in male and female rats relative to the control (*p* > 0.05).

Both a late single and double hit with alcohol significantly increased the *SREBP1c* expression in male and female rats (*p* < 0.05, Figure 3C,F). Neonatal orally administered zingerone in combination with a late single alcohol hit had no effect on *SREBP1c* expression level in male (*p* = 0.635, Figure 3C) and female (*p* = 0.960, Figure 3F) rats compared to their respective control counterparts. Neonatal orally administered zingerone in combination with a double alcohol hit had no effect on *SREBP1c* expression level in male rats (*p* > 0.05 vs. control) but it significantly increased *SREBP1c* expression in female rats (*p* = 0.005).

### 3.6. Effect of Neonatal Orally Administered Zingerone on Plasma Liver Enzyme Activities

In male and female rats, treatment regimen had no significant effect (*p* > 0.05 vs. control) on plasma AST and ALT activities (Table 2).

### 3.7. Effect of Neonatal Orally Administered Zingerone on Plasma CYP2E1 and Hepatic TBARS

**Figure 6:**
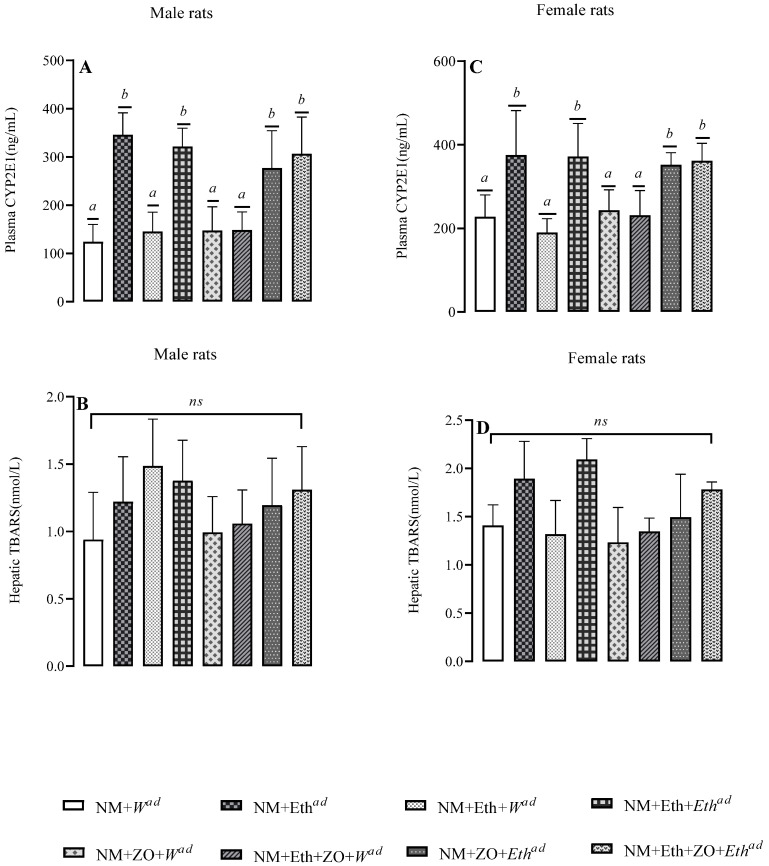
Effect of neonatal orally administered zingerone on plasma CYP2E1 (**A**,**C**) concentration and hepatic TBARS (**B**,**D**) concentrations in male (**A**,**B**) and female (D&D) rats drinking alcohol in adulthood. Data are presented as mean ± standard deviation. (*p* < 0.05). ^a,b^ = Bars means with different letters are significantly different at *p* < 0.05 Control (NM + *W^ad^*)—neonatally gavaged with nutritive milk (NM) and received plain drinking water (*W^ad^*) at stage III; late single hit (NM + *Eth^ad^*)—neonatally gavaged with NM and received ethanol (*Eth^ad^*) solution at stage III; early single hit (NM + Eth + *W^ad^*)—neonatally gavaged with ethanol in nutritive milk and received plain drinking water (*W^ad^*) at stage III; double hit (NM + Eth + *Eth^ad^*)—neonatally gavaged with Eth in nutritive milk and received *Eth^ad^* at stage III; zingerone alone (NM + ZO + *W^ad^*)—neonatally gavaged with ZO in nutritive and received plain drinking water (*W^ad^*) at stage III; early single hit with zingerone (NM + Eth + ZO + *W^ad^*)—neonatally gavaged with NM, Eth, ZO and received plain drinking water (*W^ad^*) at stage III; late single hit with zingerone (NM + ZO + *Eth^ad^*)—neonatally gavaged with NM and ZO and received *Eth^ad^* at stage III; double hit with zingerone (NM + Eth + ZO + *Eth^ad^*)—neonatally gavaged with NM, Eth and ZO and received *Eth^ad^* at stage III; ^ad^—adult treatment; NM—nutritive milk; Eth—ethanol; ZO—zingerone; W—water; *n* = 6–8 per treatment group for plasma TNF-α and IL-6. *TNF-α* and *NF-KB*; *n* = 2–3 rats per treatment group.

Ethanol consumption increased (*p* < 0.05, Figure 6A,C) CYP2E1 concentration in male and female rats that had late single hit and double hit with ethanol. Neonatal orally administered zingerone in combination with either late single hit or double hit with alcohol significantly increased CYP2E1 concentration of male and female rats relative to control. Treatment regimens had no effect on hepatic TBARS concentration in male (*p* = 0.096, Figure 6B) and female rats (*p* = 0.050, Figure 6D).

### 3.8. Effect of Neonatal Orally Administered Zingerone on Biomarkers of Inflammation

**Figure 7:**
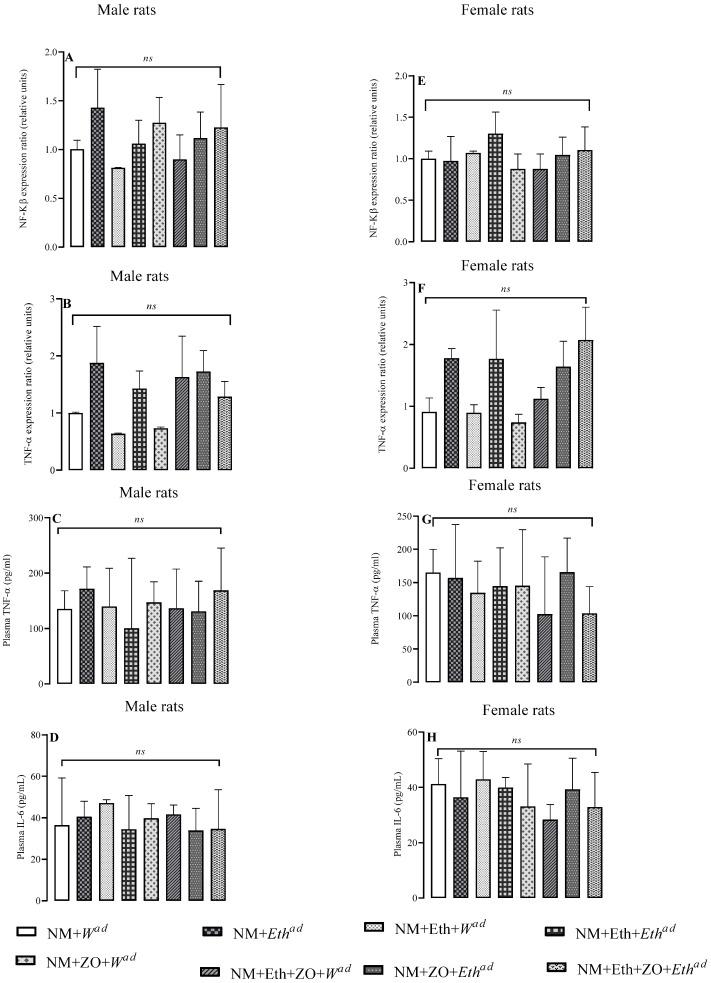
Effect of neonatal orally administered zingerone on NF-Kβ gene expression (**A**,**E**), TNF-α (**B**,**F**) gene expression, plasma TNF-α (**C**,**D**) and plasma IL-6 (**D**,**H**) concentrations in male and female rats drinking alcohol in adulthood. Data are presented as mean ± sd. ^a,a^= Bars with same letters are not significantly different at *p* > 0.05. Control (NM + *W^ad^*)—neonatally gavaged with nutritive milk (NM) and drank plain drinking water (*W^ad^*) at stage III; late single hit (NM + *Eth^ad^*)—neonatally gavaged with NM and drank ethanol (*Eth^ad^*) solution at stage III; early single hit (NM + Eth + *W^ad^*)—neonatally gavaged with ethanol in nutritive milk and drank plain drinking water (*W^ad^*) at stage III; double hit (NM + Eth + *Eth^ad^*)—neonatally gavaged with Eth in nutritive milk and drank *Eth^ad^* at stage III; zingerone alone (NM + ZO + *W^ad^*)—neonatally gavaged with ZO in nutritive and drank plain drinking water (*W^ad^*) at stage III; early single hit with zingerone (NM + Eth + ZO + *W^ad^*)—neonatally gavaged with NM, Eth, ZO and drank plain drinking water (*W^ad^*) at stage III; late single hit with zingerone (NM + ZO + *Eth^ad^*)—neonatally gavaged with NM and ZO and drank *Eth^ad^* at stage III; double hit with zingerone (NM + Eth + ZO + *Eth^ad^*)—neonatally gavaged with NM, Eth and ZO and drank *Eth^ad^* at stage III; ^ad^—adult treatment; NM—nutritive milk; Eth—ethanol; ZO—zingerone; W—water; *n* = 6–8 per treatment group for plasma TNF-α and IL-6. Gene expression for *TNF-α* and *NF-Kβ*; *n* = 2–3 rats per treatment group.

Treatment regimens had no effect (*p* > 0.05 vs. control) on plasma TNF-α and IL-6 concentrations and hepatic mRNA expression of NFK-β and TNF-α male rats and female rats (Figure 7A–H).

## 4. Discussion

In this study, we investigated the effect of neonatal orally administered zingerone on an early and late single and a double hit with alcohol on the development of alcohol-induced fatty liver disease in adulthood. A late single hit and double hit with alcohol in male and female rats resulted in an increased liver fat content accompanied by macrosteatosis, downregulation of *PPAR-α* and upregulation of *SREBP1c*. Neonatal orally administered zingerone attenuated fat accretion by preventing an upregulation in *SREBP1c* in male rats. It also mitigated hepatic steatosis and the downregulation of *PPAR-α* induced by late single or double alcohol hit in male and female rats.

Our findings showed that neonatal administered ethanol and zingerone had no effect on ethanol consumption of the rats in adulthood. Previous studies reported that exposure to alcohol during the prenatal developmental phase did not affect adolescent ethanol consumption because of aversion to alcohol developed as a result of prenatal exposure [43,44]. However, other research revealed that prenatal exposure to ethanol via food and substrate exchange between maternal and foetal blood in the placenta increases the likelihood of increasing alcohol intake in adolescence and adulthood because it enhances the brain’s reward system [3,45]. Findings from the current study suggest that the aversive as well the appetitive chemosensory stimuli to alcohol were not modified by neonatal orally administered ethanol and/or zingerone. However, we did not assess the neurochemical changes induced by the neonatal exposure to the interventions, hence there is need for further investigations into the neurochemical changes induced by exposure to alcohol and zingerone during the neonatal growth phase.

Early, late single hit and double hit with alcohol had no effect on the hepatosomatic index in the present study. In line with our finding, another research group found that alcohol consumption does not affect the hepatosomatic index of rats [46]. However, in contrast to our findings, other studies showed that alcohol consumption increased liver weight [47,48]. The studies of AL-Humadi et al., 2019 [47] and Rasineni et al., 2019 [48] made use of the Lieber–DeCarli alcohol liquid diet, which contains 35.5% compared to Labchef standard rat chow (Epol^®^, Johannesburg, South Africa) with 5% fats with alcohol solution as a drinking fluid used in this study.

A late single hit (both sexes) and double hit (females only) with alcohol increased the liver fat content. Alcohol disrupts several aspects of hepatic lipid flux that leads to lipid accumulation, including activation of *SREBP1c* to stimulate lipogenesis [49]. Studies report that alcohol causes hepatic lipid accumulation, which may result in the development of steatosis [50,51]. Hence, it was not surprising that alcohol-induced increase in liver fat content was accompanied with the formation of prominent small and large droplet macrosteatosis in both male and female rats. This observation was associated with peroxisome proliferator activator receptor-alpha (*PPAR-α*) downregulation and sterol regulatory element binding protein 1c (*SREBP1*c) upregulation. Previous studies reported that alcohol consumption caused alcohol-induced fatty liver by downregulating *PPAR-α* [52]. Furthermore, ethanol decreases the AMP-activated protein kinase (AMPK) Sirtuin (SIRT) signalling pathway and its downstream signalling proteins resulting in the upregulation of *SREBP1c* [53]. These proteins, *PPAR-α* and *SREBP1c*, are involved in regulating the hepatic fatty oxidation pathway and lipid droplet formation [26,54]. Therefore, it is also plausible that in the present study, hepatic macrosteatosis was caused by downregulation and upregulation in *PPAR-α* and *SREBP1c,* respectively.

Neonatal orally administered zingerone mitigated the alcohol-induced downregulation of hepatic *PPAR-α* expression in male and female rats but protected against *SREBP1c* upregulation in male rats only, and attenuated the accretion of liver fat and the formation of large droplet macrosteatosis, especially in the male rats. Currently, we do not have an explanation for the sexual dimorphic effect of zingerone on *SREBP1c* expression; thus, further investigation is required. Nonetheless, preceding studies have reported that sexual dimorphism in rats can be attributed to variance in rate and pattern of early development and responses to insult in male and female rats [55,56]. In vivo zingerone activates *PPAR-α* [57]. It is therefore possible that it exerted its antisteatotic effects by preventing the downregulation of *PPAR-α* Additionally, previous research had found that zingerone can suppress the expression of *SREBP1c* [33] to protect against fatty liver formation and lipid accumulation therefore our findings are in tandem with other research outcomes.

Steatosis is generally reversible after ethanol withdrawal [58]. However, we observed microsteatosis in the livers of female rats that had an early single hit with alcohol, which was neither accompanied with a significant increase in liver fat content nor an effect on the lipid regulatory genes. In line with our findings, Shen et al., 2014 [59] also reported that adult female rat offspring prenatally exposed to ethanol also exhibited microsteatosis. Presently, it is difficult to provide an explanation to these phenomena. In our recent study we observed that oral administration of ethanol during the suckling reduced the plasma triglyceride concentration of female rat pups [38]. We therefore speculate that the observed microsteatosis is likely due to the neonatal alcohol-induced export of triglycerides from the plasma to the liver.

In the current study, hepatic lobular inflammation and necrosis were not evident in the liver sections of male and female rats, suggesting that none of the interventions caused liver inflammation. Our finding is consistent with other studies that showed that in rodents the consumption of alcohol only did not induce significant hepatic inflammation and necrosis [60], except under prolonged (29 weeks) consumption of 40% ethanol [61]. Additionally, the rats only developed simple steatosis without a progression to alcoholic steatohepatitis. Simple steatosis is not associated with inflammation [62,63,64], though given more time, this could have progressed to steatohepatitis. Plasma AST and ALT activities are considered surrogate biomarkers for liver injury [65]. Ethanol consumption that caused hepatocyte inflammation and necrosis was shown to be associated with increased plasma AST and ALT activity [66] (Huang et al., 2010) and Yamasaki et al., 2019 [67] reported no change in AST and ALT activity without inflammation and necrosis. However, Radic et al., 2019 [68] observed that despite causing hepatic focal inflammation and mild necrosis, the consumption of ethanol did not affect plasma AST and ALT activity of rats suggesting poor correlation between liver enzyme activity and damage [69]. Our findings showed similarities in histological findings on hepatocytes with no evidence of necrosis and inflammation which explains similarities in plasma ALT and AST concentration of the rats since there was no liver cell damage. The present data indicate that eight weeks of ethanol consumption by the rats was not sufficient to induce liver cell injury. Importantly, we show that neonatal orally administered zingerone did not cause liver inflammation and injury and this finding is in agreement with its previously reported effects in adult rats [36,70,71].

Our findings showed that ethanol consumption in the late single and double hit groups elevated CYP2E1 concentration of the rats. CYP2E1 catalyses the metabolism of alcohol [27] in the liver. Chronic ethanol consumption induces CYP2E1 activity in order to facilitate ethanol metabolism [27]. Additionally, ethanol has been reported to induce and stabilize CYP2E1 proteins post-translationally [72]. Contrary to our finding, Kolata et al., 2020 [73] did not observe an increased CYP2E1 protein in male Wistar rats that consumed 10% ethanol solution for 6 weeks. This variance might be because we used 20% ethanol, a concentration that was 100% higher. Furthermore, the current study used Sprague–Dawley rats, which could have also brought differences in rat strain responses. In the current study, we observed that neonatal orally administered zingerone did not affect the alcohol-induced increase in CYP2E1 proteins. Acute pharmacokinetic study of zingerone in rats via mass spectrophotometry demonstrated that it does not significantly affect of the microsomal content of P450 enzymes [74]. Therefore, zingerone cannot be used to promote alcohol abstinence as the induction of CYP2E1 is associated with greater tolerance to high ethanol intake [27].

The induction of CYP2E1 is associated with alcohol-induced oxidative stress via the generation of acetaldehyde and increased production of free radical species, which can form adducts with DNA and cause oxidative tissue injury [27]. However, our findings recorded similar hepatic TBARS concentration across treatment regimens, which suggests no initiation of significant lipid peroxidation in both male and female rats. Our findings are in agreement with the works of Kołota et al., 2020 [73] and Radic et al., 2019 [68] but at variance with Teare et al., 1994 [75] and Keegan et al., 1995 [61] due possibly to the difference in experimental duration and the development of more advanced stages of ALD. Therefore, our findings corroborate the hypothesis that oxidants from CYP2E1 play a minor role in the mechanisms involved in the early stages of ALD [76], although extended alcohol consumption or a more sensitive biomarker such as F2-isoprostanes might have yielded a significant result, as TBARs are not considered sensitive and reliable biomarkers of lipid peroxidation due to their reactivity and metabolism [77]. Late single and double hit with alcohol increased hepatic TBARs by 44.1% and 52.0%, respectively, in the female rats. However neonatal orally administered zingerone reduced TBARS by 27.1% and 23.7% in the late single and double alcohol hit groups, respectively. Our study corroborates previous reports that indicate that zingerone markedly reduces lipid peroxidation via its free radical scavenging ability [36,78]. While it has been previously reported that zingerone can reduce high-fat diet induced steatosis and its associated inflammation, what is not known is its ability to programme for long-term protection against alcohol-induced fatty liver disease.

## 5. Conclusions

The present study showed that a late and double hit with alcohol in rats resulted in the development of AFLD, characterised by small and large droplet macrosteatosis, with a downregulation in *PPAR-α* and an upregulation in *SREBP1c* without significant inflammatory changes or elevation in liver enzymes. Alcohol fatty liver disease renders the liver susceptible to toxic effects of alcohol and other insults. However, neonatal orally administered zingerone attenuated AFLD in a sex-dependent manner. Zingerone can therefore be strategically administered in the neonatal phase as a potential prophylactic agent for its beneficial effects against AFLD and ability to blunt the development ALD, and lessen the burden of ALD on the healthcare system. This information should be taken into consideration when developing guidelines regarding alcohol consumption during breastfeeding. Additionally, this study recommends fortifying diets of breastfeeding mothers with ginger, a rich source of zingerone, for its prophylactic benefits against diseases.

## Figures and Tables

**Table 1:** Effect of neonatal orally administered zingerone on absolute liver mass and hepatosomatic indices in male and female rats drinking alcohol in adulthood.

Treatment Group	Males		Females	
	Liver Mass (g)	Hepatosomatic Index (%)	Liver Mass (g)	Hepatosomatic Index (%)
NM + *W^ad^*	14.19 ± 1.76 ^a^	2.99 ± 0.24 ^a^	8.06 ± 0.49 ^ab^	2.92 ± 0.10 ^a^
NM + *Eth^ad^*	13.12 ± 1.21 ^a^	2.96 ± 0.18 ^a^	7.73 ± 0.62 ^ab^	2.78 ± 0.12 ^a^
NM + Eth + *W^ad^*	13.85 ± 2.39 ^a^	2.97 ± 0.25 ^a^	8.46 ± 0.95 ^a^	2.90 ± 0.18 ^a^
NM + Eth + *Eth^ad^*	12.76 ± 1.57 ^a^	2.84 ± 0.16 ^a^	7.56 ± 0.61 ^ab^	2.80 ± 0.21 ^a^
NM + ZO + *W^ad^*	14.19 ± 1.76 ^a^	2.97 ± 0.13 ^a^	7.81 ± 0.98 ^ab^	2.82 ± 0.17 ^a^
NM + Eth + ZO + *W^ad^*	13.70 ± 1.29 ^a^	2.94 ± 0.12 ^a^	8.29 ± 0.48 ^ab^	2.89 ± 0.18 ^a^
NM + ZO + *Eth^ad^*	11.73 ± 1.25 ^a^	2.77 ± 0.21 ^a^	7.28 ± 0.52 ^b^	2.75 ± 0.11 ^a^
NM + Eth + ZO + *Eth^ad^*	12.30 ± 0.95 ^a^	2.80 ± 0.19 ^a^	7.38 ± 0.41 ^b^	2.82 ± 0.10 ^a^

Data are presented as mean ± sd. ^a,b^ = within column means with different letters significantly different at *p* < 0.05. Control (NM + *W^ad^*)—neonatally gavaged with nutritive milk (NM) and drank plain drinking water (*W^ad^*) at stage III; late single hit (NM + *Eth^ad^*)—neonatally gavaged with NM and drank ethanol (*Eth^ad^*) solution at stage III; early single hit (NM + Eth + *W^ad^*)—neonatally gavaged with ethanol in nutritive milk and drank plain drinking water (*W^ad^*) at stage III; double hit (NM + Eth + *Eth^ad^*)—neonatally gavaged with Eth in nutritive milk and drank *Eth^ad^* at stage III; zingerone alone (NM + ZO + *W^ad^*)—neonatally gavaged with ZO in nutritive and drank plain drinking water (*W^ad^*) at stage III; early single hit with zingerone (NM + Eth + ZO + *W^ad^*)—neonatally gavaged with NM, Eth, ZO and drank plain drinking water (*W^ad^*) at stage III; late single hit with zingerone (NM + ZO + *Eth^ad^*)—neonatally gavaged with NM and ZO and drank *Eth^ad^* at stage III; double hit with zingerone (NM + Eth + ZO + *Eth^ad^*)—neonatally gavaged with NM, Eth and ZO and drank *Eth^ad^* at stage III; ^ad^—adult treatment; NM—nutritive milk; Eth—ethanol; ZO—zingerone; W—water; *n* = 6–8 per treatment group.

**Table 2:** Effect of neonatal orally administered zingerone on plasma liver enzyme activities in adult male and female rats drinking alcohol in adulthood.

Treatment Group	Males		Females	
	AST(U/L)	ALT (U/L)	AST (U/L)	ALT (U/L)
NM + *W^ad^*	247.8 ± 155.5 ^a^	61.8 ± 11.3 ^a^	147.2 ± 35.9 ^a^	57.0 ± 19.5 ^a^
NM + *Eth^ad^*	298.8 ± 130.4 ^a^	114.0 ± 47.2 ^a^	168.2 ± 46.6 ^a^	62.8 ± 22.2 ^a^
NM + Eth + *W^ad^*	210.6 ± 81.8 ^a^	96.2 ± 36.2 ^a^	161.3 ± 77.0 ^a^	50.8 ± 19.5 ^a^
NM + Eth + *Eth^ad^*	156.4 ± 46.2 ^a^	81.0 ± 30.3 ^a^	200.7 ± 74.6 ^a^	69.5 ± 32.6 ^a^
NM + ZO + *W^ad^*	183.3 ± 45.7 ^a^	70.3 ± 21.2 ^a^	145.0 ± 58.3 ^a^	51.8 ± 18.9 ^a^
NM + Eth + ZO + *W^ad^*	224.6 ± 115.3 ^a^	79.8 ± 28.1 ^a^	121.4 ± 28.3 ^a^	43.0 ± 7.3 ^a^
NM + ZO + *Eth^ad^*	207.1 ± 102.6 ^a^	75.8 ± 25.8 ^a^	117.6 ± 14.1 ^a^	46.8 ± 9.4 ^a^
NM + Eth + ZO + *Eth^ad^*	228.7 ± 75.81 ^a^	95.8 ± 28.7 ^a^	165.0 ± 69.5 ^a^	46.83 ± 7.5 ^a^

Data are presented as mean ± sd. ^a,a^ = Within column means that do not differ significantly at *p* > 0.050. Control (NM + *W^ad^*)—neonatally gavaged with nutritive milk (NM) and drank plain drinking water (*W^ad^*) at stage III; late single hit (NM + *Eth^ad^*)—neonatally gavaged with NM and drank ethanol (*Eth^ad^*) solution at stage III; early single hit (NM + Eth + *W^ad^*)—neonatally gavaged with ethanol in nutritive milk and drank plain drinking water (*W^ad^*) at stage III; double hit (NM + Eth + *Eth^ad^*)—neonatally gavaged with Eth in nutritive milk and drank *Eth^ad^* at stage III; zingerone alone (NM + ZO + *W^ad^*)—neonatally gavaged with ZO in nutritive and drank plain drinking water (*W^ad^*) at stage III; early single hit with zingerone (NM + Eth + ZO + *W^ad^*)—neonatally gavaged with NM, Eth, ZO and drank plain drinking water (*W^ad^*) at stage III; late single hit with zingerone (NM + ZO + *Eth^ad^*)—neonatally gavaged with NM and ZO and drank *Eth^ad^* at stage III; double hit with zingerone (NM + Eth + ZO + *Eth^ad^*)—neonatally gavaged with NM, Eth and ZO and drank *Eth^ad^* at stage III; ^ad^—adult treatment; NM—nutritive milk; Eth—ethanol; ZO—zingerone; W—water; *n* = 5–6 per treatment group.

## Data Availability

Data is contained within the article or Supplementary Material.

## Supplementary Materials

The following supporting information can be downloaded at: https://www.mdpi.com/article/10.3390/metabo13020167/s1, Table S1: Primer sequences; Table S2: Effect of neonatal zingerone on ethanol consumption in alcohol-exposed rats in adult (A) male and (B) female rats.
