# Supplementary material for: Neonatal Orally Administered Zingerone Attenuates Alcohol-Induced Fatty Liver Disease in Experimental Rat Models
Source: Metabolites. 2023 Jan 23;13(2):167. doi: 10.3390/metabo13020167 (PMC9966972; doi:10.3390/metabo13020167)
Supplement: Supplementary file 1 [file metabolites-13-00167-s001.zip › metabolites-2110946-supplementary.pdf]

Results

Table S1. Primer sequences

| Primer Name | Sequence 5′→3′ |
|---|---|
| SREBP-1C F | CGACACCACCAGCATCAACCACG |
| SREBP-1C R | GCAGCCCDATTCATCAGCCAGACC |
| BETAACTIN F | GCTAACAGTCCGCCTAGAAGCA |
| BETAACTIN R | GTCATCACCATCGGGCAATGAG |
| PPAR-α F | GATACCACTATGGAGTCCACGCA |
| PPAR-α R | GCCGAAAGAAGCCCTTG |
| NF-Kβ F | CGTGAAGTATTCCCAGGTTTG |
| NF-Kβ R | TGGGGGAAAACTCATCAAAG |
| TNF-α F | GAAGTTCCCAAATGGCCTCC |
| TNFα R | GTGAGGGTCTGGGCCATAGA |

Table S2. Effect of neonatal zingerone on ethanol consumption in alcohol-exposed rats in adult (A) male and (B) female rats

| A. ***Males*** | | | | |
|---|---|---|---|---|
| | Weekly ethanol consumption (g/100g/body mass) | | | |
| Week | NM+Eth | NM+Eth+Eth | NM+ZO+Eth | NM+Eth+ZO+Eth |
| Wk 1 | 3.46±0.91[a] | 2.64±0.53[a] | 2.98±0.91[a] | 2.77±0.42[a] |
| Wk 2 | 5.20±0.68[b] | 5.41±1.40[b] | 5.50±1.13[b] | 5.37±1.46[b] |
| Wk 3 | 7.40±1.64[b] | 7.16±1.42[b] | 7.59±1.89[b] | 7.64±1.77[b] |
| Wk 4 | 9.81±0.82[b] | 8.30±1.40[b] | 8.48±1.07[b] | 9.35±1.99[b] |
| Wk 5 | 9.63±0.81[b] | 8.59±2.44[b] | 8.94±1.61[b] | 8.95±1.48[b] |
| Wk 6 | 9.79±0.9[b] | 7.86±1.68[b] | 8.99±1.54[b] | 8.85±1.55[b] |
| Wk 7 | 9.39±3.47[b] | 8.09±2.37[b] | 8.86±3.30[b] | 8.55±1.85[b] |
| Wk 8 | 10.16±1.18[b] | 8.68±1.38[b] | 9.26±1.49[b] | 9.25±1.32[b] |
| Mean | 8.10±2.52 | 7.02±2.09 | 7.57±2.22 | 7.59±2.34[b] |

| A. ***Females*** | | | | |
|---|---|---|---|---|
| | Weekly ethanol consumption (g/100g/body mass) | | | |
| Week | NM+Eth | NM+Eth+Eth | NM+ZO+Eth | NM+Eth+ZO+Eth |
| Wk 1 | 3.79±0.94[a] | 4.15±1.01[a] | 4.61±0.57[a] | 4.50±1.26[a] |
| Wk 2 | 6.68±1.32[b] | 8.06±2.47[b] | 7.52±2.78[b] | 7.64±1.82[b] |
| Wk 3 | 9.89±1.49[b] | 12.53±8.81[b] | 10.62±3.24[b] | 9.76±1.60[b] |
| Wk 4 | 11.16±2.82[b] | 13.43±5.53[b] | 10.05±1.60[b] | 10.90±1.96[b] |
| Wk 5 | 11.99±6.65[b] | 10.72±1.51[b] | 12.11±2.7[b] | 11.42±3.88[b] |
| Wk 6 | 10.81±2.70[b] | 10.37±0.90[b] | 10.23±2.54[b] | 10.51±1.14[b] |
| Wk 7 | 10.58±2.77[b] | 9.21±1.95[b] | 11.46±4.18[b] | 10.04±2.07[b] |
| Wk 8 | 11.72±1.74[b] | 12.49±1.81[b] | 12.31±1.55[b] | 11.97±1.93[b] |
| Mean | 9.58±2.87 | 10.12±3.01 | 9.86±2.61 | 9.59±2.44 |

Data is presented as mean ± standard deviation. [ab] = preceding week's ethanol intake was significantly lower at p< 0.05. **NM + W =** gavaged with 10 ml/kg body mass per day nutritive milk during suckling + plain drinking water in adulthood; **NM+Eth+ W =** gavaged with 10 ml/kg body mass per day nutritive milk and Eth during suckling + plain drinking water in adulthood; **NM+Eth** = gavaged with 10 ml/kg body mass per day nutritive milk during suckling + Eth solution in adulthood; **NM + Eth+ Eth** = gavaged with 10 ml/kg body mass per day nutritive milk and Eth during suckling + Eth solution in adulthood; **NM+ ZO + W** = gavaged with 10 ml/kg body mass per day nutritive milk and ZO during suckling + plain drinking water in adulthood; **NM+Eth+ZO+W** = gavaged with 10 ml/kg body mass per day nutritive milk, Eth and ZO during suckling + plain drinking water in adulthood; **NM+ZO+Eth** = gavage with 10 ml/kg body mass per day nutritive milk and ZO during suckling + Eth solution in adulthood; **NM+Eth+ZO+ Eth** = gavage with 10 ml/kg body mass per day nutritive milk, Eth and ZO during suckling + Eth solution in adulthood n = 6-8 per treatment group
